# iTRAQ-Based Proteomics Analysis Reveals the Effect of Rhubarb in Rats with Ischemic Stroke

**DOI:** 10.1155/2018/6920213

**Published:** 2018-07-16

**Authors:** Xiangping Lin, Tao Liu, Pengfei Li, Zehui He, Yuanyuan Zhong, Hanjin Cui, Jiekun Luo, Yang Wang, Tao Tang

**Affiliations:** ^1^Laboratory of Ethnopharmacology, Institute of Integrated Traditional Chinese and Western Medicine, Xiangya Hospital, Central South University, 410008 Changsha, China; ^2^Department of Gerontology, Traditional Chinese Medicine Hospital Affiliated to Xinjiang Medical University, 830000 Urumqi, China

## Abstract

*Background. *Rhubarb, a traditional Chinese medicine, promotes viscera and remove blood stasis. Rhubarb is skilled in smoothening meridians, improving blood circulation which exhibits better efficacy on cerebral ischemic stroke. In this study, we aimed to analyze the underlying mechanisms of rhubarb which treated rats of middle cerebral artery occlusion (MCAO) model according to an iTRAQ-based proteomics and bioinformatics analysis. 30 rats were randomly allocated into three groups including sham group (SG), model group (MG), and rhubarb group (RG). Rhubarb group was given a gavage of rhubarb decoction at dose of 3 g/kg and the remaining groups were prepared with normal saline by gavage. Rats from MG and RG were induced into MCAO model. The effects of rhubarb were estimated by Modified Neurological Severity Score (mNSS) and cerebral infarct volume. The brain tissues were measured via the quantitative proteomic approach of iTRAQ coupled to liquid chromatography-tandem mass spectrometry (LC-MS/MS). Furthermore, the bioinformatics analysis of overlapping differentially expression proteins (DEPs) was conducted by DAVID, KEGG, and Cytoscape. Specific selective DEPs were validated by Western blotting. Rats treated with rhubarb after MCAO showed a significant reduction on mNSS and cerebral infarct volume compared with MG. In MG versus SG and RG versus MG, we identified a total of 4578 proteins, of which 287 were DEPs. There were 76 overlapping DEPs between MG versus SG and RG versus MG. Through bioinformatics analysis, 14 associated pathways were searched including cGMP-PKG signaling pathway, tuberculosis, synaptic vesicle cycle, amyotrophic lateral sclerosis, long-term potentiation, and so on. 76 overlapping DEPs mainly involved synaptic vesicle cycling biological processes based on GO annotation. Further, the selective overlapping DEPs were verified at the protein level by using Western blotting. Our present study reveals that rhubarb highlights promising neuroprotective effect. Rhubarb exerts novel therapeutic action via modulating multiple proteins, targets, and pathways.

## 1. Introduction

Stroke is the second leading cause of death and has remained one of the biggest killers globally in the last 15 years [1, 2]. Approximately 11.6 million ischemic stroke events are in low- and middle-income countries [3]. In 2013, there was an estimated 10.3 million new strokes, 67% of which were ischemic stroke in the United States [4]. The prevalence rate of ischemic stroke nearly doubled from 1990 to 2013 globally [5]. In view of the above facts, the government from each country tends to develop the prevention of ischemic stroke as a national priority.

Ischemic stroke generally leads to a cascade of pathological mechanisms, contributing to the irreversible tissue damage including mitochondrial dysfunction, cerebral edema, cerebral hemorrhage, and neuronal death [6, 7]. Pathogenically, ischemic stroke accounts for 85% of all conditions caused by vessel occlusions [8]. These patients were provided antiplatelet or anticoagulant drugs at acute ischemic stroke onset [9]. Unfortunately, these drugs could increase the risk of fatal or disabling intracranial hemorrhage, which offset any benefits in current ischemic stroke [10]. Thus, the novel therapeutic agents with safety and efficacy have attracted much attention of neuroscientists and doctors.

The clinical applications and experimental studies have reported that traditional Chinese medicines including Radix/rhizome notoginseng extract, Buyang Huanwu decoction, and Danhong injection are ascendant against convalescent ischemic stroke [11–13]. Nevertheless, few studies and clinical trials focus on the earlier phase of acute therapy for ischemic stroke. According to the traditional Chinese medicine theory, the core of ischemic stroke refers to binding phlegm-heat and blood stasis syndrome. In the light of this, rhubarb (*Rheum palmatum *L. or* Rheum tanguticum *Maxim, dahuang in China) is skilled in smoothening meridians, improving blood circulation which exhibits better efficacy on cerebral ischemia [14]. Modern studies show that rhubarb protects the blood brain barrier and provides neuroprotective effect via an antioxidative molecular mechanism in brain injury [15, 16]. However, there is no explicit mechanism responsible by rhubarb treatment for ischemic stroke.

Due to the effects of herbal medicine via multiple targets and multidirectional treatments, single pharmacological action cannot unfold the effects of rhubarb to treat ischemic stroke. Within recent years, omics-based methods are applied to uncover the multipathways of herbal medicine for disease treatment [17]. The proteomics is rapidly emerging as an essential tool to gain insights into complex systems at the level of protein expression. It has been widely used to detect novel targets, study interaction of proteins, and discover the associated mechanisms of action in the life sciences [18, 19]. The main method for proteomics study includes iTRAQ which combines with multidimensional liquid chromatography (LC) and mass spectroscopic (MS) analysis [20]. It has become a crucial tool in quantitative proteomics due to its high throughput, sensitivity, and accuracy [21, 22]. This approach enables comparison between normal and disease samples to uncover the dysregulation of proteins [23]. The iTRAQ has been frequently utilized in the studies of traditional Chinese herbs such as Gastrodia elata blume and Pseudostellaria heterophylla [24, 25]. Hence, it is feasible to reveal the multiple targeting effects of rhubarb in treating acute ischemic stroke-based on quantitative proteomics.

In this study, the iTRAQ-based quantitative proteomic approach was applied to identify disease relevant proteins in rat model of ischemic stroke. We next used the bioinformatics analyses including KEGG, DAVID, and Cytoscape to exhibit the potential targets and molecular signal pathways of rhubarb to treat acute ischemic stroke. The further verifications were conducted by Western blotting. The present study could help to facilitate the rhubarb as a potential therapeutic agent for acute ischemic stroke.

## 2. Materials and Methods

### 2.1. Experimental Animals

Male Sprague-Dawley (SD) rats weighing 220–240g were purchased from Laboratory Animal Centre of Central South University (CSU) and allowed to acclimatize for at least 5 days. The environmental conditions were maintained in specific pathogen free grade. 30 rats were randomly allocated into three groups including sham group (SG), model group (MG), and rhubarb group (RG). RG was given a gavage of rhubarb decoction at dose of 3 g/kg and the rest groups were prepared with normal saline by gavage. The animal procedures were approved and verified by the Animal Ethics Committee of CSU.

### 2.2. Chemicals and Drugs

The antibodies of Synapsin-1 and *β*-tubulin were from Proteintech Group (Chicago, USA). ERK1/2 antibody was from the American Abcam Company (Cambridge, UK). All the other reagents were of analytical grade or better. Rhubarbs (voucher specimen no. 2015101605, Gansu, China) were purchased from Pharmacy of Traditional Medicine of Xiangya Hospital Central South University (Changsha, Hunan). Dried rhubarb was crushed into powder and soaked in distilled water for 30 min. They were boiled for 10 min at 100°C. The extraction yield was around 1:12 w/v. The decoction was stored under 4°C. The method of identifying and quantifying the principal components on rhubarb decoction had been reported in our previous study [26].

### 2.3. Establishment of the Middle Cerebral Artery Occlusion (MCAO) Model

Rat was produced by transient intracranial MCAO, as formerly described [27]. Rats were placed in a supine position. The right common carotid artery (CCA), the right external carotid artery (ECA), and the right internal carotid artery (ICA) were exposed and isolated. A 4-0 nylon monofilament suture (Beijing Cinontech Co., Ltd., Beijing, China) coated with 1% poly-L-lysine was inserted from the CCA to the ICA and gently advanced to occlude the origin of the middle cerebral artery (MCA). The suture had reached the circle of Willis. After 2h of occlusion, the MCA-suture was carefully removed for reperfusion.

The suture was withdrawn to allow reperfusion 24h after MCAO. Rats in SG received all the surgical procedures except that the arteries were not occluded. SG and MG were given physiological saline under the same conditions. After assessment of the neurological deficits, rats were sacrificed. All surgical operations were finished in a sterile environment with body temperatures maintained at 37± 0.5°C.

### 2.4. Neurological Evaluation and TTC Staining

Neurological functional assessments were performed at 24 h after MCAO using Modified Neurological Severity Score (mNSS) [28, 29]. Neurological function was graded on a scale of 0 to 18 (normal score, 0; maximal deficit score, 18). The more severe the injury, the higher the scores of the rats. The dead rats would be excluded. The brain tissues were rapidly removed and frozen for 20 min at -20°C. Frozen cerebra were sliced into 2 mm thick coronal section and incubated in 2,3,5-triphenyltetrazolium chloride (TTC) at 37°C for 30 min. Then the infarct volume of each section was measured with an image analysis system (Image-ProPlus 6.0, Media Cybernetics, MD, USA). % Infarct volume = (white infarct area ×thickness) / (whole slice area × thickness) ×100% [30].

### 2.5. Proteomics

#### 2.5.1. Protein Extraction

Briefly, the total protein from each cerebral tissue sample was ground to powder with liquid nitrogen and dissolved in lysis solution. The cell lysate was centrifuged at 12,000 rpm for 20 min and the supernatant was collected. All extraction steps were carried out at 4°C.

#### 2.5.2. iTRAQ Labeling

From each sample, 200 mg of protein was reduced, alkylated, and trypsin-digested overnight at 37°C. The labeling was performed according to the manufacturer's instructions for the iTRAQ Reagents 8-plex kit (AB Sciex, Foster City, CA, USA). Samples from MG were labeled with tag 113. The SG was labeled with 114. RG was labeled with tag 116.

#### 2.5.3. Peptide Fractionation with Strong Cation Exchange (SCX)

For SCX chromatography using the Shimadzu LC-20AD HPLC Pump system (Shimadzu Corp, Kyoto, Japan), the peptides from digestion were reconstituted with buffer A (20mM HCOONH4, PH 10) and loaded onto a 2 × 150 mm Gemini-NX C18 SCX column containing 3-*μ*m particles (Phenomenex) with buffer B (20 mM HCOONH4 80% acetone PH 10). Elution was monitored by measuring absorbance at 214nm/280 nm. The flow rate was 200*μ*l/min. Eluted peptides were collected as 24 fractions and vacuum-dried. Each final fraction was resuspended in 50% trifluoroacetic acid.

#### 2.5.4. LC-MS/MS Analysis

The peptides were analyzed on a Q Exzctive mass spectrometer (Thermo Scientific) and identified using the Thermo Dionex Ultimate 3000 RSLC nanosystem. The binary buffer system was consisting of 0.1% formic acid (buffer A) and 84% CAN in 0.1% formic acid (buffer B). Each fraction was dissolved in 0.1% formic acid and 2% ACN. The supernatant was loaded onto a PepMap C18 RP column (2 *μ*m, 75 *μ*m × 150 mm, 100 A). The peptides separated under a 65min linear gradient from 4% buffer B to 90% buffer B at a constant flow rate of 300 nl/min.

The eluted peptides were directly detected by Q Exactive online. MS data were acquired in a data-dependent mode using a top 20 high-energy collisional dissociation (HCD) method, which dynamically chose the most abundant precursor ions from the scan range (350–1800 m/z). The dynamic exclusion was set to 40 s, and the automatic obtaining control (AGC) target value was 3E6. The underfill ratio was defined as 0.1%.

#### 2.5.5. Proteomics Data Analysis

The obtained raw data were searched with Protein Pilot Software (AB Sciex, Foster, CA, USA; version 5.0.1) against the UniProt rat database. The search parameters were the following: iTRAQ 8 plex peptide labeled was selected for sample type and trypsin was selected as the digestion enzyme. Biological modifications were selected as ID focus. Only proteins with unused value more than 1.3 were considered for further analysis (equal to protein confidence was set at 95%). The data were based on a false discovery rate (FDR) ≤1% confidence for protein identification.

### 2.6. Bioinformatics Analysis

The networks were generated and analyzed by using Cytoscape 3.5.1. We used ClueGO, a plugin of Cytoscape, to constitute the network for Gene Ontology (GO) mapping and annotation. Kyoto encyclopedia of genes and genomes (KEGG) pathways analysis was performed using DAVID Bioinformatics Resources v6.8 (https://david.ncifcrf.gov/).* P *value ≤0.05 was considered to indicate significant pathways.

### 2.7. Western Blotting

The frozen cerebral tissues (n=4 per group) were collected in ice-cold lysis buffer. The lysates were centrifuged at 12000 g for 10 min at 4°C. The protein concentration was measured by the BCA protein assay kit (Well Biotechnology Company, Changsha, China). The proteins were loaded and separated by 10% sodium dodecyl sulfate polyacrylamide gel electrophoresis (SDS-PAGE) and transferred to polyvinylidene fluoride (PVDF) membranes. The membrane was blocked at room temperature with a 5% milk-Tris-buffered saline and Tween 20 (TBST) solution for 1 h and then incubated with the anti-Syn1 (1:2000), anti-ERK1/2 (1:10000), and anti-*β*-tubulin (1:1000) overnight at 4°C. After being washed thrice with TBST (15 min each), the membranes were incubated with horseradish peroxidase-labeled secondary antibody (1:6000, Proteintech Group, Chicago, USA). After incubation finished, the membranes were washed three times (15 min each) and visualized using enhanced chemiluminescence detection system (ECL; Thermo Fisher Scientific, Shanghai, China) for 5 min before exposure to X-ray film. Western blotting bands were scanned and analyzed with UN-Scan-It 6.1 software (Silk Scientific, Inc., Orem, UT) [31].

### 2.8. Statistics Analysis

All data are expressed as mean ± standard deviation (SD) and evaluated by one-way ANOVA with SPSS version 18.0. The results in each group were compared by t-test. A* p* value<0.05 was considered to be statistically significant.

## 3. Results

### 3.1. Rats Treated with Rhubarb Show Attenuated Infarction Volume Rate and Improved Neurological Deficits

**Figure 1:**
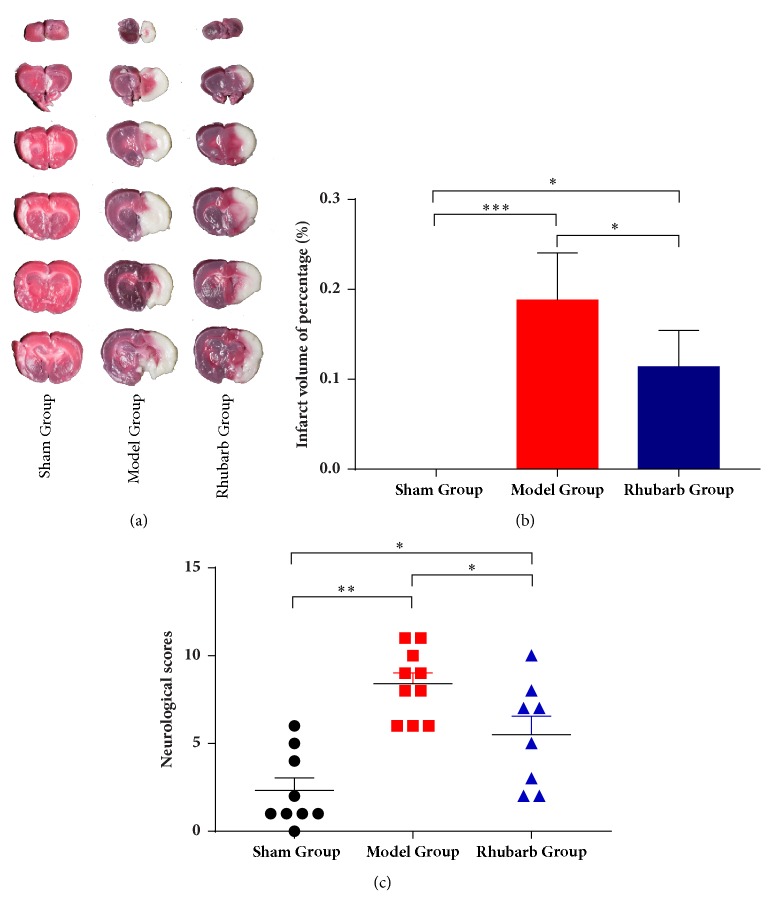
(a) Representative TTC stained brain sections of sham group (n=3), model group (n=5), and rhubarb group (n=5) treated ischemic animals. (b) Infarct volume was determined 24 h after MCAO. (c) There were significant differences in neurological deficits at 24 h after MCAO between sham group (n=9), model group (n=10), and rhubarb group (n=8). Data are presented as mean ± SD. ∗*p*<0.05, ∗∗*p*<0.01, and ∗∗∗*p*<0.001.

After 24 h reperfusion, 3 rats were dead and neurological assessments were conducted for the remaining rats. TTC staining visually showed the histological changes of cerebral tissues (Figure 1(a)). SG appeared uniform red in color, indicating no infarction. Infraction volume of percentage showed a dramatic increase in MG (18.82% ± 5.23%) compared with SG (Figure 1(b)). Rhubarb significantly reduced acute infarction volume ratio (11.38% ± 4.04%) after 24 h reperfusion. As can be seen in Figure 1(c), obvious differences in regard to MCAO abnormality were exhibited between SG and MG (*p*<0.01). Rats treated with rhubarb showed a significant reduction of neurological deficits on mNSS compared with MG (*p*<0.05).

### 3.2. Quantitative Proteomic Analysis of Brains with MCAO Rats

In order to comprehensively understand the mechanism of rhubarb in ischemic stroke, iTRAQ analysis was performed on the protein extracts from rat brain with or without rhubarb treatment. After merging data from the respective replicates, 4578 proteins were identified and showed different expression (fold change ≥ 1.2, FDR ≤ 1%). Only the upregulated proteins with 1.5 ≤ an average ratio change ≤ 5 or downregulated proteins with an average ratio change ≤ 0.667. Peptides (95%) ≥ 2 and unused value ≥ 2 in both replicates were considered to be significantly expressed and used for the following analysis.

**Figure 2:**
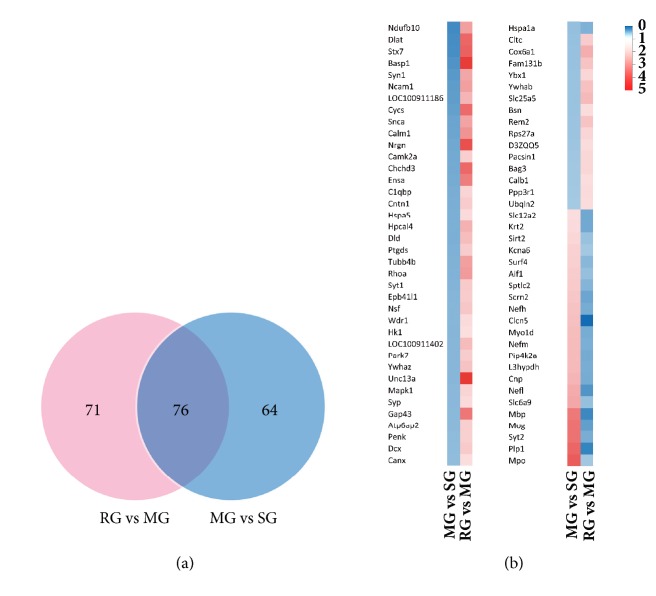
The number of differentially expressed proteins (DEPs) and their overlap was exhibited in Venn diagram (a). 76 proteins with upregulated and downregulated expressions between MG versus SG and RG versus MG was showed in our study (b). Significant changes in the protein ratios were changed at least 1.5-fold.

In MG versus SG and RG versus MG, 287 proteins were differentially expression proteins (DEPs). There were 147 DEPs in RG versus MG and 140 DEPs in MG versus SG, respectively.  Figure 2(a) illustrated the proteins between the RG versus MG and MG versus SG by a Venn diagram. Only 64 DEPs were changed in MG versus SG uniquely, whereas 71 DEPs were regulated by rhubarb treatment alone. 76 overlapping DEPs showed altered protein expression level in both experimental sets and rhubarb group (Figure 2(b)).  Table 1 summarized detailed information of 76 overlapping DEPs, including the UniProt accession number, gene name, peptides, fold change, and unused value. 53 overlapping DEPs were specifically upregulated, while 23 overlapping DEPs were downregulated after rhubarb treatment. Further, overlapping DEPs were subjected to functional analysis with reference to KEGG database.

### 3.3. KEGG Database Analysis

**Figure 3:**
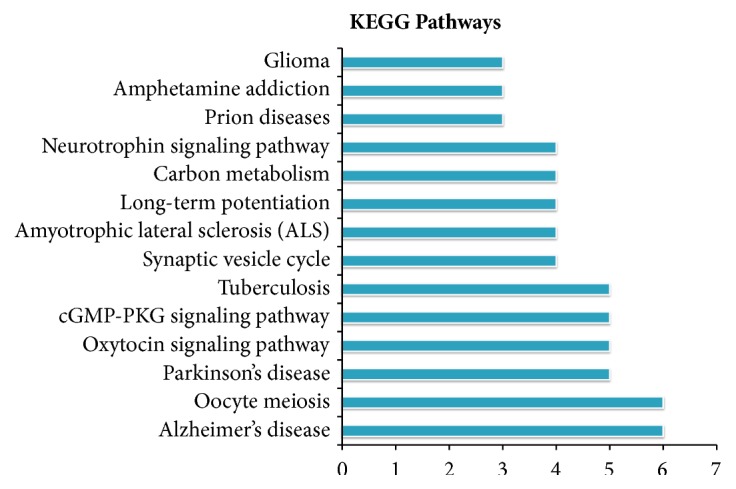
KEGG pathway analysis of overlapping DEPs identified by iTRAQ analysis.

The analysis was performed by matching the overlapping DEPs to the proteins annotated with KEGG pathway database via DAVID Bioinformatics Resources v6.8. The identified pathways were potentially affected by the modification of the abundance of proteins in the rat brain. Then 75 overlapping DEPs were identified and 1 overlapping DEP was not by DAVID. Among these overlapping DEPs, only 46 (56%) overlapping DEPs had a KEGG Orthology ID and were involved in 26 pathways. 14 pathways were statistically significant with* p* value < 0.05. The KEGG analysis results were shown in Figure 3 and Table 2. There are almost all closely related to Alzheimer's disease, oocyte meiosis, Parkinson's disease, oxytocin signaling pathway, cGMP-PKG signaling pathway, tuberculosis, synaptic vesicle cycle, amyotrophic lateral sclerosis (ALS), long-term potentiation (LTP), carbon metabolism, neurotrophin signaling pathway, prion diseases, amphetamine addiction, and glioma.

### 3.4. Protein-Protein Interaction (PPI) Network Analysis

**Figure 4:**
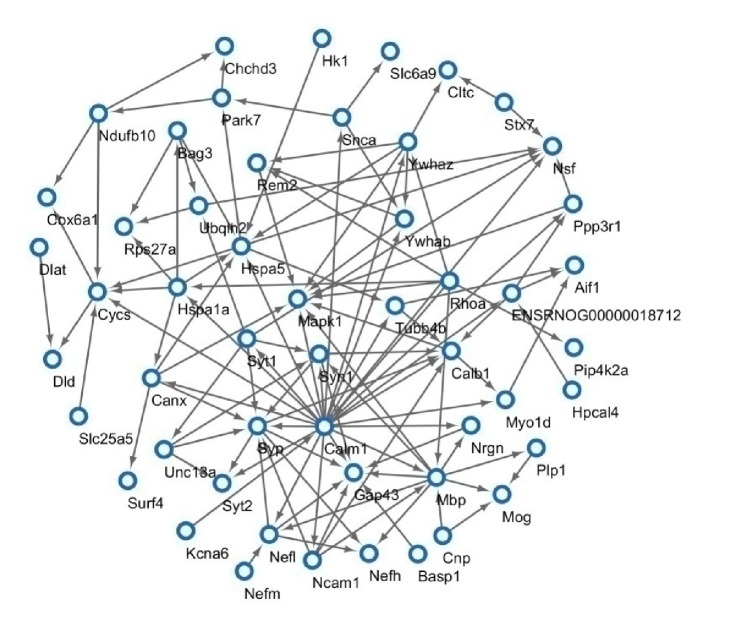
Protein network connectivity of identified proteins in MCAO rat with or without rhubarb.

Further PPI network analysis was conduct by Cytoscape. PPI network (Figure 4) showed that several significant proteins might function in response to ischemic stroke. Of these 75 identified overlapping DEPs, a total of 52 were linked into a protein interaction network and 23 did not show any link. The network analysis revealed potential relationship between Mapk1, Syn1, Calm1, Syp, Gap43, Hspa5, Mbp, and so on. The network view summarized the network of predicted associations for particular proteins. It provided us a crucial modeling scaffolds and data reduction way which made us got insight into the potential mechanisms of rhubarb in brain.

### 3.5. GO Analysis

**Figure 5:**
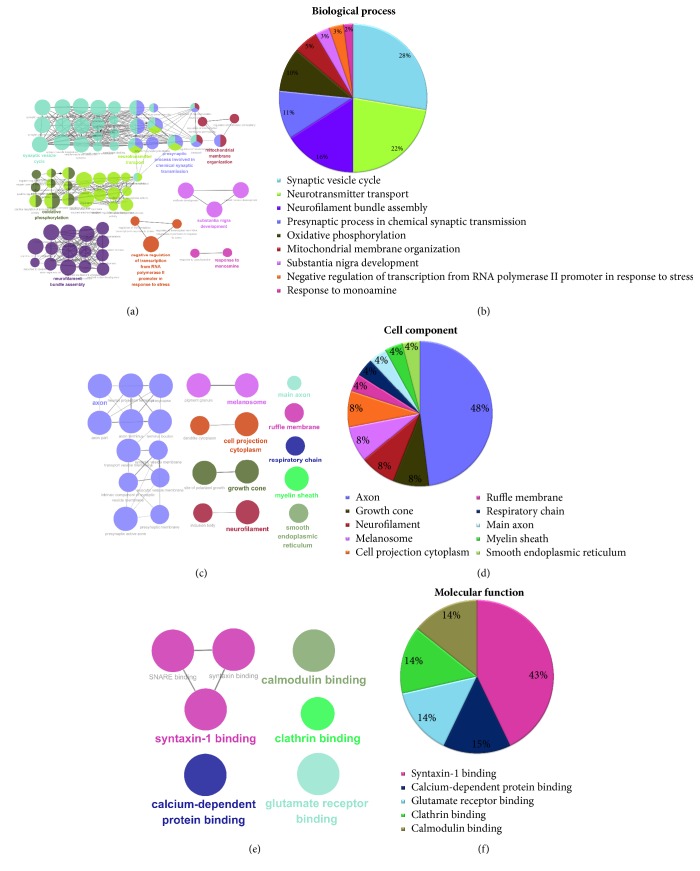
GO annotation analysis of the overlapping DEPs. The color had a one-to-one correspondence between (a), (c), (e) and (b), (d), (f), respectively. ((a) and (b)) GO annotation enriched by ClueGO according to biological process. ((c) and (d)) GO annotation enriched by ClueGO according to cellular components. ((e) and (f)) GO annotation enriched by ClueGO molecular function. Functionally grouped network with terms linked by nodes, and functionally related groups were partially overlapped; the node size represented the term enrichment significance.

To detect the potential role of rhubarb in MCAO rats, we allocated 76 overlapping DEPs to biological process, cell component, and molecular function based on GO annotation. Biological processes that overlapping DEPs participated were representatively involved in synaptic vesicle cycle (28%), neurotransmitter transport (22%), neurofilament bundle assembly (16%), presynaptic process involved in chemical synaptic transmission (11%), oxidative phosphorylation (10%), mitochondrial membrane organization (5%), substantia nigra development (3%), negative regulation of transcription from RNA polymerase II promoter in response to stress (3%), and response to monoamine (2%) (Figures 5(a) and 5(b)).

Cell component suggested that proteins were located-representatively in the axon (48%), growth cone (8%) neurofilament (8%), melanosome (8%), cell projection cytoplasm (8%), Ruffle membrane (4%), respiratory chain (4%), main axon (4%), myelin sheath (4%), and smooth endoplasmic reticulum (4%) (Figures 5(c) and 5(d)).

Most overlapping DEPs for the molecular functions classification were associated with syntaxin-1 binding (43%), calcium-dependent protein binding (15%), glutamate receptor binding (14%), clathrin binding (14%), and calmodulin binding (14%) (Figures 5(e) and 5(f)).

### 3.6. Rhubarb Increased the Expression Level of ERK1/2 and Syn1

**Figure 6:**
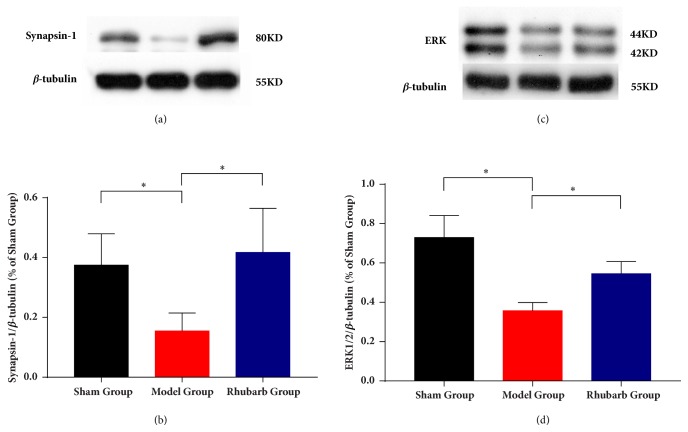
Western blotting analyses of Syn1 and ERK1/2. Proteins were examined for four times and normalized to *β*-tubulin levels (loading control) for quantitative analysis. The levels of protein expression are presented as means ± SD. *∗p*<0.05 compared with model.

To confirm the results from the iTRAQ analysis, protein expression levels were evaluated for the 2 identified proteins Mapk1 (also means ERK2) and Synapse1 (Syn1) using Western blotting (n=4, each group) (Figure 6). The results showed that the ratio of Syn1/*β*-tubulin and ERK1/2/*β*-tubulin were decreased in both brain tissues with CIR. Meanwhile, the protein expressions of Syn1 and ERK were increased significantly compared with MG. In comparison of SG, the increased protein relative expression of rhubarb group had not statistic difference in both Syn1 and ERK. In addition, quantitative protein levels by iTRAQ demonstrated the same trend for Syn1 and ERK in Western blotting.

## 4. Discussion

In this study, we first confirmed the effective treatment of rhubarb which significantly reduced infarction volume ratio and neurological assessments on MCAO model rats. Next, we performed a quantitative proteomics and bioinformatics analysis to explore the disease-related protein abundance. Further comparing RG with MG, Western blotting identified the changes of candidate proteins which were in accord with the results of proteomics. Our study may suggest that rhubarb is expected to be a novel drug for ischemic stroke.

To the best of our knowledge, few studies reveal the mechanism of rhubarb on acute ischemic stroke. To address this concern, the iTRAQ-based proteomics analysis was utilized to exploit multiple targets and pathways following ischemic stroke in rats model of MCAO. As a result, we identified a total of 4578 proteins, of which 287 were DEPs. Among the three groups, there were 76 overlapping DEPs. 76 overlapping DEPs were part of altered protein after cerebral ischemia and rhubarb treatment interfered with these 76 proteins at the same time. Furthermore, there were 53 upregulated proteins and 23 downregulated proteins after rhubarb treatment. Then for these 76 overlapping DEPs a series of bioinformatics analysis were conducted. By GO annotation, it involves a variety of biological processes, including synaptic vesicle cycle and neurotransmitter transport, among others both in MG and RG.

In KEGG pathways analysis, it exhibited that cerebral ischemia and rhubarb treatment involve oxytocin signaling pathway, cGMP-PKG signaling pathway, synaptic vesicle cycle, and LTP in top 14 pathways. Oxytocin (OT) which is a typical hormone responds to many pathological conditions following ischemia-reperfusion. According to altered expression patterns of GABAA receptor subunit and the kinetics of GABA-induced chloride ion in*ﬂ*ux, OT conferred neuroprotection against ischemic stroke [32]. OT also could inhibit the calpain to improve morphology damage in neurons after ischemic stroke [33]. Through the activating cGMP-dependent protein kinas (PKG) and resultant phosphorylation of cellular proteins, cGMP play a role of a second messenger which exerts most of its action in central nervous system [34]. The activation of the cGMP-PKG signaling pathway enhanced LTP at thalamic inputs to the lateral amygdala (LA). It was suggested that synaptic plasticity and fear memory consolidation might be promoted via NO-cGMP-PKG in the LA and in part by activating the ERK/MAPK signaling cascade [35]. Rhubarb may exert a multitarget and multipathway regulation mechanism through these overlapping DEPs in ischemic stroke.

Therefore, we chose Mapk1 and Syn1 for confirming the reproducibility of our studies and the excellent quality of our data. In iTRAQ analysis and the results of Western blotting, Mapk1 and Syn1 were obviously upregulated after rhubarb treatment. Mapk1 (also known as MAPK1, p42MAPK) and Syn1 have important roles in synaptic transmission and plasticity [36, 37].The synaptic damage and plasticity could affect the development of treatments to facilitate rehabilitation of patients with ischemic cerebral damage [38]. Animal evidence indicates that it opens a time-limited window of neuroplasticity following a stroke which is the greatest gains in recovery [39].

Mapk signaling cascade is critical for both the memory consolidation and the long-term neuronal plasticity. ERK1 and ERK2 are activated in neurons in response to excitatory glutamatergic signaling, which controls many forms of synaptic plasticity [40–42]. Studies also demonstrate that ERK signaling plays an important role in the induction of LTP which is an activity-dependent strengthening of synaptic efficacy [43, 44]. ERK1/2 has also been shown to phosphorylate Syn1 which is a major substrate in nerve terminals [45]. Syn1, one of the synapsins family, is synaptic vesicle phosphoproteins regulating neural development, plasticity, and synaptic transmission [37, 46]. The ERK1/2-dependent phosphorylation of Syn1 is stimulated hippocampus-dependent learning. The increase of neurotransmitter release selectively in LTP facilitated enhancements in hippocampus-dependent learning [47]. Furthermore, Syn1 is required by ERK1/2-dependent synaptic plasticity [48]. Knocking out Syn1 will inhibit the enhancements of learning, presynaptic plasticity, and LTP [49]. Study also shows that Syn1 is the main presynaptic effector for certain forms of LTP triggered by the activation of the H-ras-Mapk pathway [43]. By activating the ERK1/2-Syn1 signaling pathway, the collagen nanofibrous scaffolds promoted to the presynaptic maturation [50]. Our Western blotting results suggested that ERK1/2 and Syn1 were significantly increased by the treatment of rhubarb compared with the model group. There are robustly associated with the synaptic vesicle cycle and presynaptic process involved in chemical synaptic transmission regulated by rhubarb following ischemic stroke. Thus, it is concluded that rhubarb may be essential for the development of synaptic plasticity via ERK1/2-Syn1 signal pathway.

Rhubarb is one of the important Chinese herbal medicines. Its major active components including rhein and chrysophanol are the anthraquinones which exist in combined (glycosides) and free (aglycones) forms. The rhubarb and its extracts are involved in decreasing the content of NO in brain tissue and antianoxia on ischemic stroke [51, 52]. The study reports that anthraquinones present protective effect against focal cerebral ischemia via resisting the aggregation and adhesion of platelet or decreasing plasminogen and fibrinogen [53]. Rhubarb aglycone presents protective effect according to improving cerebral infraction and neuronal apoptosis in cerebral ischemia/reperfusion [54]. Chrysophanol, an active component of rhubarb aglycones, protects the damage of brain tissues following ischemic stroke via inhibiting the activation of inflammasome [55]. These studies and our research support that rhubarb may represent a potential neuroprotection modulating multiple proteins and pathways for ischemic stroke. In a further study, we should investigate more upstream or downstream molecular mechanisms from the DEPs in detail.

## 5. Conclusions

The present study reveals that rhubarb highlights a promising neuroprotective effect on acute MCAO rats. Rhubarb exerts the treatment via modulating multiple proteins, targets, and pathways in a rat model of ischemic stroke. By the verification of molecular biology technique, the rhubarb exhibits essential impact on synaptic plasticity. Our study might provide a novel therapeutic agent of cerebral ischemic stroke.

## Figures and Tables

**Table 1:** Quantitative information of the overlapping DEPs in RG versus MG.

**No.**	**Uniprot Accession**	**Gene name**	**Unused**	%**Cov**	**Protein identity**	**Peptides**	**AVG.**
				**(95)**			
						**(95%)**	
**1**	G3V7C6	Tubb4b	2	76.64	Tubulin beta-4B chain	274	2.43
**2**	Q9WTY2	Rem2	2.04	4.98	GTP-binding protein REM 2	2	1.88
**3**	Q04940	Nrgn	4.01	46.15	Neurogranin	7	3.81
**4**	P10818	Cox6a1	4.08	27.93	Cytochrome c oxidase subunit 6A1, mitochondrial	3	2.21
**5**	P04094	Penk	4.1	11.15	Proenkephalin-A	3	1.70
**6**	P22057	Ptgds	4.23	29.63	Prostaglandin-H2 D-isomerase	6	1.76
**7**	P35332	Hpcal4	6.4	49.21	Hippocalcin-like protein 4	12	2.15
**8**	P60841	Ensa	7.12	47.11	Alpha-endosulfine	7	2.99
**9**	Q3ZAV2	Ybx1	7.86	27.30	Nuclease-sensitive element-binding protein 1	4	1.60
**10**	G3V997	Dcx	8.55	20.82	Neuronal migration protein doublecortin	9	1.80
**11**	Q6AXS4	Atp6ap2	9.02	16.00	Renin receptor	8	1.73
**12**	P37377	Snca	9.85	80.71	Alpha-synuclein	22	2.37
**13**	O35796	C1qbp	10.07	28.32	Complement component 1 Q subcomponent-binding protein, mitochondrial	8	1.63
**14**	B7X6I3	Cend1	10.49	56.38	C38 protein	19	2.00
**15**	F8WFH6	Fam131b	11.76	20.28	Protein FAM131B	7	1.89
**16**	Q5U2U8	Bag3	12	17.42	Bcl2-associated athanogene 3	9	1.60
**17**	P35213	Ywhab	12.04	78.46	14-3-3 protein beta/alpha	65	1.92
**18**	O70257	Stx7	12.21	39.85	Syntaxin-7	13	3.47
**19**	A0A0G2K0M8	Ncam1	12.94	50.30	Neural cell adhesion molecule 1	77	2.44
**20**	P07825	Syp	13.95	40.07	Synaptophysin	19	1.55
**21**	P07171	Calb1	16.2	41.38	Calbindin	16	1.51
**22**	D3ZUX5	Chchd3	16.88	35.68	MICOS complex subunit	13	3.34
**23**	P62982	Rps27a	17.28	54.49	Ubiquitin-40S ribosomal protein S27a	23	1.62
**24**	D4A0T0	Ndufb10	18.05	61.93	Protein Ndufb10	15	2.43
**25**	Q09073	Slc25a5	18.08	61.07	ADP/ATP translocase 2	46	2.03
**26**	A0A0H2UHV6	Ppp3r1	18.44	61.25	Calcineurin subunit B type 1	17	1.55
**27**	D4A5L9	LOC679794	18.53	71.43	Protein LOC679794	24	3.31
**28**	P61589	Rhoa	19.6	64.77	Transforming protein RhoA	24	2.52
**29**	F1M378	Unc13a	20.94	6.01	Protein unc-13 homolog A	11	4.26
**30**	O88767	Park7	21.87	91.53	Protein deglycase DJ-1	22	1.76
**31**	D4AA63	Ubqln2	24.33	27.12	Protein Ubqln2	22	1.53
**32**	P14604	Echs1	24.5	53.45	Enoyl-CoA hydratase, mitochondrial	20	2.09
**33**	P08461	Dlat	27.5	35.60	Dihydrolipoyllysine-residue acetyltransferase component of pyruvate dehydrogenase complex, mitochondrial	25	3.38
**34**	P63086	Mapk1	31.61	49.16	Mitogen-activated protein kinase 1	26	1.63
**35**	Q6P6R2	Dld	33.08	54.03	Dihydrolipoyl dehydrogenase, mitochondrial	31	1.98
**36**	Q05175	Basp1	34.26	86.82	Brain acid soluble protein 1	82	4.18
**37**	P62161	Calm1	34.4	89.93	Calmodulin	80	2.64
**38**	P11275	Camk2a	35.04	64.44	Calcium/calmodulin-dependent protein kinase type II subunit alpha	76	1.73
**39**	P35565	Canx	35.34	37.39	Calnexin	31	1.50
**40**	P07936	Gap43	37.7	84.51	Neuromodulin	48	3.07
**41**	Q9Z0W5	Pacsin1	38.67	56.46	Protein kinase C and casein kinase substrate in neurons protein 1	50	1.61
**42**	P21707	Syt1	40.02	45.61	Synaptotagmin-1	37	1.79
**43**	Q5RKI0	Wdr1	42.43	46.37	WD repeat-containing protein 1	33	1.53
**44**	P63102	Ywhaz	43.94	85.31	14-3-3 protein zeta/delta	77	1.90
**45**	P06761	Hspa5	57.41	55.20	78 kDa glucose-regulated protein	57	1.55
**46**	P05708	Hk1	76.09	47.17	Hexokinase-1	80	1.50
**47**	Q63198	Cntn1	77.28	48.29	Contactin-1	77	1.74
**48**	D3ZMI4	Epb41l1	82.1	34.24	Band 4.1-like protein 1	52	1.77
**49**	P09951	Syn1	91.64	72.30	Synapsin-1	152	2.34
**50**	F1LQ81	Nsf	104.81	69.22	N-ethylmaleimide sensitive fusion protein, isoform CRA_b	86	1.84
**51**	D3ZQQ5	Dnm1	112.88	68.06	Dynamin-1	107	1.52
**52**	G3V984	Bsn	155.27	32.42	Protein bassoon	111	1.53
**53**	F1M779	Cltc	171.28	58.69	Clathrin heavy chain	213	1.76
**54**	A0A0G2K839	Clcn5	2	4.41	Chloride channel protein	3	0.02
**55**	G3V8L6	Kcna6	2.13	6.04	Potassium voltage-gated channel subfamily A member 6	2	0.65
**56**	D3ZV91	L3hypdh	4	7.34	Protein L3hypdh	2	0.48
**57**	F1LSV4	Sptlc2	4	5.18	Protein Sptlc2	3	0.52
**58**	A0A0G2K1A2	Mpo	4.01	3.76	Protein Mpo	3	0.65
**59**	P28572	Slc6a9	4.94	8.62	Sodium- and chloride-dependent glycine transporter 1	5	0.60
**60**	P55009	Aif1	5.28	23.13	Allograft inflammatory factor 1	5	0.60
**61**	Q6AYR8	Scrn2	5.8	13.00	Secernin-2	6	0.43
**62**	A0A0G2JWX4	Krt2	6.06	9.36	Keratin, type II cytoskeletal 2 epidermal	4	0.45
**63**	G3V6M3	Syt2	6.27	29.38	Synaptotagmin II	17	0.48
**64**	Q9R0I8	Pip4k2a	7.18	22.66	Phosphatidylinositol 5-phosphate 4-kinase type-2 alpha	14	0.46
**65**	Q7TP91	Surf4	10	9.55	Ab1-205	9	0.55
**66**	P0DMW1	Hspa1b	16.76	41.34	Heat shock 70 kDa protein 1B	73	0.51
**67**	P60203	Plp1	17.43	31.41	Myelin proteolipid protein	50	0.20
**68**	Q63345	Mog	18.16	36.73	Myelin-oligodendrocyte glycoprotein	14	0.39
**69**	A0A0G2JWM2	Sirt2	31.35	52.32	NAD-dependent protein deacetylase sirtuin-2	34	0.61
**70**	Q63357	Myo1d	31.36	21.27	Unconventional myosin-Id	27	0.48
**71**	E9PTX9	Slc12a2	34.28	21.28	Protein Slc12a2	26	0.44
**72**	P02688	Mbp	35.6	66.67	Myelin basic protein	88	0.23
**73**	F1LRZ7	Nefh	54.4	41.26	Neurofilament heavy polypeptide	55	0.47
**74**	P19527	Nefl	55.56	58.49	Neurofilament light polypeptide	87	0.32
**75**	P13233	Cnp	62.44	74.52	2′,3′-cyclic-nucleotide 3′-phosphodiesterase	83	0.47
**76**	P12839	Nefm	79.88	49.88	Neurofilament medium polypeptide	86	0.49

**Table 2:** KEGG pathways associated with the 76 overlapping DEPs by DAVID.

**Term**	**Count**	**PValue**	**Genes**
rno04114: Oocyte meiosis	6	2.47E-04	P63102, P62161, P35213, A0A0H2UHV6, P63086, P11275
rno05010: Alzheimer's disease	6	0.00251	P62161, P10818, D4A0T0, A0A0H2UHV6, P63086, P37377
rno05012: Parkinson's disease	5	0.008091	O88767, Q09073, P10818, D4A0T0, P37377
rno04921: Oxytocin signaling pathway	5	0.009657	P61589, P62161, A0A0H2UHV6, P63086, P11275
rno04022: cGMP-PKG signaling pathway	5	0.011878	P61589, P62161, Q09073, A0A0H2UHV6, P63086
rno05152: Tuberculosis	5	0.014949	P61589, P62161, A0A0H2UHV6, P63086, P11275
rno05014: Amyotrophic lateral sclerosis (ALS)	4	0.002947	P19527, P12839, A0A0H2UHV6, F1LRZ7
rno04721: Synaptic vesicle cycle	4	0.004142	F1LQ81, P21707, F1M779, F1M378
rno04720: Long-term potentiation	4	0.004732	P62161, A0A0H2UHV6, P63086, P11275
rno01200: Carbon metabolism	4	0.025472	P05708, Q6P6R2, P14604, P08461
rno04722: Neurotrophin signaling pathway	4	0.028277	P61589, P62161, P63086, P11275
rno05020: Prion diseases	3	0.012162	P06761, A0A0G2K0M8, P63086
rno05031: Amphetamine addiction	3	0.044482	P62161, A0A0H2UHV6, P11275
rno05214: Glioma	3	0.045744	P62161, P63086, P11275

## Data Availability

All MS data have been deposited in the PRIDE Archive with the dataset identifiers PXD006437 (http://www.ebi.ac.uk/pride/archive/login, Username: reviewer58335@ebi.ac.uk, Password: IXAJht8T).
